# Secret of Comfort

**Published:** 1854-12

**Authors:** 


Secret of Comfort.—Though sometimes small evils, like invisible insects, inflict pains, and a small hair may stop a vast machine, yet the chief secret of comfort lies in not suffering trifles to vex one, and prudently cultivating an undergrowth of small pleasures, since very few great ones, alas! are let on long leases.—*Sharp's Essays.*

